# The Malaysian HEalth and WellBeing AssessmenT (MyHEBAT) Study Protocol: An Initiation of a National Registry for Extended Cardiovascular Risk Evaluation in the Community

**DOI:** 10.3390/ijerph191811789

**Published:** 2022-09-19

**Authors:** Al’aina Yuhainis Firus Khan, Anis Safura Ramli, Suraya Abdul Razak, Noor Alicezah Mohd Kasim, Yung-An Chua, Ahmad Zia Ul-Saufie, Mohd Amin Jalaludin, Hapizah Nawawi

**Affiliations:** 1 Institute of Pathology, Laboratory and Forensic Medicine (I-PPerForM), Universiti Teknologi MARA, Sungai Buloh 47000, Selangor, Malaysia; 2 Departments of Primary Care Medicine, Faculty of Medicine, Universiti Teknologi MARA, Sungai Buloh 47000, Selangor, Malaysia; 3 Cardio Vascular and Lungs Research Institute (CaVaLRI), Universiti Teknologi MARA, Sungai Buloh 47000, Selangor, Malaysia; 4 UiTM Al-Sultan Abdullah Hospital, Puncak Alam 42300, Selangor, Malaysia; 5 Department of Pathology, Faculty of Medicine, Sungai Buloh Campus, Universiti Teknologi MARA, Jalan Hospital, Sungai Buloh 47000, Selangor, Malaysia; 6 Faculty of Computer and Mathematical Sciences, Universiti Teknologi MARA, Shah Alam 40450, Selangor, Malaysia; 7 Department of Otorhinolaringology, Faculty of Medicine, University of Malaya, Kuala Lumpur 50603, Wilayah Persekutuan Kuala Lumpur, Malaysia

**Keywords:** cardiovascular disease, CVD risk factor, risk categorisation, familial hypercholesterolaemia

## Abstract

Cardiovascular disease (CVD) has been a burden to many developing countries for decades, including Malaysia. Although various steps have been taken to prevent and manage CVD, it remains the leading cause of morbidity and mortality. The rising prevalence of CVD risk factors such as hypertension, hypercholesterolaemia, diabetes, overweight and obesity is the main driving force behind the CVD epidemic. Therefore, a nationwide health study coined as the Malaysian Health and Wellbeing Assessment (MyHEBAT) was designed. It aimed to investigate the prevalence of CVD and the associated risk factors in the community across Malaysia. The MyHEBAT study recruited participants (18–75 years old) through community health screening programmes from 11 states in Malaysia. The MyHEBAT study was further divided into two sub-studies, namely, the Cardiovascular Risk Epidemiological Study (MyHEBAT-CRES) and the MyHEBAT Familial Hypercholesterolaemia Study (MyHEBAT-FH). These studies assessed the prevalence of CVD risk factors and the prevalence of FH in the community, respectively. The data garnered from the MyHEBAT study will provide information for healthcare providers to devise better prevention and clinical practice guidelines for managing CVD in Malaysia.

## 1. Introduction

For decades, cardiovascular disease (CVD) has remained the leading cause of death globally due to the rising epidemic of CVD risk factors and increasing ageing population [1,2]. Studies estimated that CVD was accountable for 422.7 million cases and 17.9 million deaths in 2015, and it is projected to cause 23.6 million deaths per year by 2030 [3,4,5]. In Malaysia, CVD mortality and morbidity burden have been escalating over the past three decades. The Malaysian Ministry of Health Report showed CVD remained the leading cause of death from the 1980s until today [6,7]. In 2019, 15.0% of 109,164 medically certified deaths were caused by coronary artery disease (CAD), and this is 2.5 times more than all cancer-related death combined [8,9]. Risk factors for CVD are classified into modifiable and unmodifiable risk factors. The established modifiable risk factors are tobacco use, high blood pressure, high cholesterol, obesity, diabetes, consumption of alcohol, unhealthy diet, physical inactivity, overweight and obesity [1,2,3,6]. Meanwhile, the unmodifiable risk factors for CVD are gender, race and positive family history [10,11].

Familial hypercholesterolaemia (FH) is a common autosomal dominant genetic disorder that leads to severe elevation of low-density lipoprotein cholesterol (LDL-C) in the blood at a young age [12,13,14], which subsequently accelerates the development of atherosclerotic cardiovascular disease (ASCVD). Despite the significant role of FH in causing ASCVD, FH is still underdiagnosed, and many FH patients with an established diagnosis are still undertreated [15,16,17]. Despite the estimate that 320,000 individuals have FH in Malaysia out of the total population of 32 million, the detection rate is extremely low (0.5%) [18,19]. It has been postulated that hypercholesterolaemic patients without FH have a 6-fold higher CAD risk compared to the normal population, but hypercholesterolaemic individuals with molecularly confirmed FH have a staggering 22-fold increased CAD risk [20]. Early initiation of lipid-lowering medication in FH patients at a young age has been demonstrated to be effective at reducing the LDL-C exposure such that the CAD risk is reduced to just as low as in the non-FH population during old age [21]. Therefore, identification of FH is important in distinguishing those who require timely and aggressive lipid-lowering medication, thereby preventing the loss of treatment opportunity.

## 2. The Rationale of the Study

The rising prevalence of modifiable and unmodifiable CVD risk factors, including FH, among the Malaysian population, has been a cause of great concern [22]. Therefore, there is a need for conducting a persistent epidemiological study to establish a database consisting of nationwide big data on CVD risk factors and FH among the Malaysian population. The database will serve as a complimentary reference for the National Health and Morbidity Survey (NHMS) conducted by the Ministry of Health Malaysia. According to the NHMS, the prevalence of hypertension increased from 34.6% in 2006 to 35.3% by 2015, and was the highest contributor to CVD risk, which has a prevalence of 32.7% (5.8 million) [11,23,24]. In 2019, the NHMS also reported that the prevalence of diabetes increased from 11.2% in 2011 to 13.4% in 2015, and that of hypercholesterolaemia increased from 35.1% (2011) to 47.7% (2015) [7].

Over the past five decades, the Malaysian population experienced rapid urbanisation and changes in their diet and physical activity. In the 1950s and 1960s, the majority of the Malaysian population were blue-collar workers (farmers and labourers), who were physically active. Rapid economic development over the last three decades offered more white-collar jobs (professional, desk, managerial, or administrative work), which has driven Malaysians to adopt a sedentary lifestyle [25,26]. This phenomenon has resulted in an increasing trend in morbidity-inducing behaviours, including consumption of alcohol, eating an unhealthy diet (frequent consumption fatty food, salty food, deep-fried food or snacks, meat and poultry), physical inactivity and smoking. In addition, apart from lifestyle changes, a study has demonstrated that gender played a role in contributing to CVD risk factors: smokers and alcohol drinkers were predominantly males; and physical inactivity and obesity were significantly higher among females [27]. In 2019, it was estimated that more than 27,200 Malaysian deaths annually were related to smoking [7]. Physical inactivity that causes abdominal obesity has a prevalence of 51.2%, the most common risk factor among the study population [11]. In another recent study, elderly females with higher incomes were more likely to be obese and have diabetes as well [28,29].

Since 2017, the Malaysian government has been actively promoting a healthy lifestyle through advertisement and awareness programmes, informing the public and patients of the importance of knowing their CVD risks [30]. The National Strategic Plan for Non-Communicable Diseases (NSP-NCD) 2016–2025 outlined five main objectives: reducing modifiable risk factors, prevention and control of NCD, promoting high-quality research for prevention of NCD and evaluating the progress of prevention NCD are among the objectives [31]. However, despite these efforts, the prevalence of CVD risk factors among Malaysians continues to increase. Epidemiological data on Malaysia’s health and well-being related to CVD are limited and restricted to certain states or areas only. Similarly, the epidemiological data of FH in terms of its role as a CVD risk factor are still under-reported in Malaysia [32].

Moreover, from the economic viewpoint, uncontrolled NCD and its risk factors will cause an increase in the number of people with disabilities who are unable to produce goods and services for Malaysia, resulting in economic loss due to rising healthcare costs and declining labour productivity. Reports on the impact of NCD and their risk factors on Malaysia’s gross domestic product in 2020 disclosed that CVD and diabetes topped the list of NCD which incurred productivity loss and increased the disease costs burden for individuals and the government [33]. The same report explained the loss of productivity due to death of workers for CVD in 2017 was RM 4.281 billion (60.7%) from the total NCD, suggesting that the government is at loss in managing the CVD burden.

## 3. Malaysian Health and Wellbeing Assessment (MyHEBAT)

The Malaysian Health and Wellbeing Assessment (MyHEBAT) study is a national-level health screening programme consisting of joint efforts from multiple medical specialties, which have been assembled to tackle Malaysian health and well-being issues closely related to CVD. MyHEBAT investigators consist of experts from several research institutes and Malaysian public universities who are contributing their expertise to aid the process of organising the health screening programmes, sample collection, providing logistics support and research facilities, and to conduct data analysis and report writing. The MyHEBAT study is further divided into two subgroups, namely, the MyHEBAT-Cardiovascular Risk Epidemiological Study (CRES) and the MyHEBAT Familial Hypercholesterolaemia (FH) Study.

### 3.1. MyHEBAT Aims and Objectives

The ultimate aims of MyHEBAT study are to (1) determine the prevalences of CVD risk factors and FH in the Malaysian population; (2) establish a database consisting of nationwide big data on CVD risk factors and FH in the Malaysian population. The protocol to achieve these objectives is outlined in this paper.

### 3.2. MyHEBAT Cardiovascular Risk Epidemiological Study (MyHEBAT-CRES)

The cardiovascular disease prevalence in Malaysia is mainly being reported by the NHMS. Other small studies had limited amounts of data, covering only specific geographical area, and did not represent the Malaysian population as a whole. In addition to that, the data from the blood specimen in NHMS studies only measured the total cholesterol (TC) and did not include the entire lipid profile, i.e., triglyceride (TG), high-density lipoprotein cholesterol (HDL-C) and LDL-C, which have valuable information for determining the CVD risk factor stratification [7,34]. Hence, the MyHEBAT-CRES study aims to bridge the gap by providing comprehensive epidemiological data in order to guide prevention strategies and accurate patient management.

MyHEBAT-CRES recruited participants through nationwide sampling which covered urban/rural areas in 11 states of Malaysia. Instead of targeting participants with a specific disease or condition, MyHEBAT-CRES recruited Malaysian participants with or without known health status, since several studies in Malaysia indicated that Malaysians lacked awareness of their health status and were unaware of which risk factors contributed to CVD [5,7,27,35].

Additionally, variables correlated with Malaysian sociodemographic and Malaysian lifestyle will be investigated in depth. The degree of association between variables and CVD will be determined as well. A study in 2015 reported that having multiple risk factors was associated with even greater risk for CVD compared to having less risk factor [27]. Hence, the MyHEBAT-CRES study will assess which combination of multiple CVD risk factors contributed to mortality and morbidity prevalence in the Malaysian population. In addition, the participants will be stratified using various CVD risk tools, namely, the Framingham Risk Score—Cardiovascular Disease (FRS-CVD), the 2016 European Society of Cardiology/European Atherosclerosis Society (ESC/EAS) Guidelines and the 2019 ESC/EAS guideline [36,37,38,39].

### 3.3. MyHEBAT Familial Hypercholesterolaemia Study (MyHEBAT-FH)

Currently, FH is still under-detected and under treated in Malaysia, where it could lead to premature CAD. FH causes prolonged exposure of the vasculature to high LDL-C levels, which results in the development of atherosclerotic lesions in the heart, brain and peripheral arteries [40]. Previous studies reported that FH patients have better quality of life when treated with statin, as its use showed a significant reduction of CAD risk by 76% [18,41]. Furthermore, a study reported that early statin treatment can delay or prevent the onset of CAD [21].

Since the MyHEBAT-FH study recruited participants among the community, we expected that participants would be unaware of their FH status. Hence, one of the main aims of MyHEBAT-FH study is to report the prevalence of FH in Malaysia. The data were standardised as one of the crucial sources of FH database in Malaysia with sociodemographic background and CVD risk factors to understand FH in Malaysia. In addition, the MyHEBAT-FH study aims to characterise FH categories using four types of clinical diagnostic criteria, namely, Dutch Lipid Clinic Network Criteria (DLCN), Simon Broome’s Register Criteria (SB), United States Make Early Diagnosis to Prevent Early Deaths Criteria (USMEDPED) and Japanese Familial Hypercholesterolaemia Management Criteria (JFHMC) [42].

## 4. Methods

### 4.1. Study Design and Population

**Figure 1:**
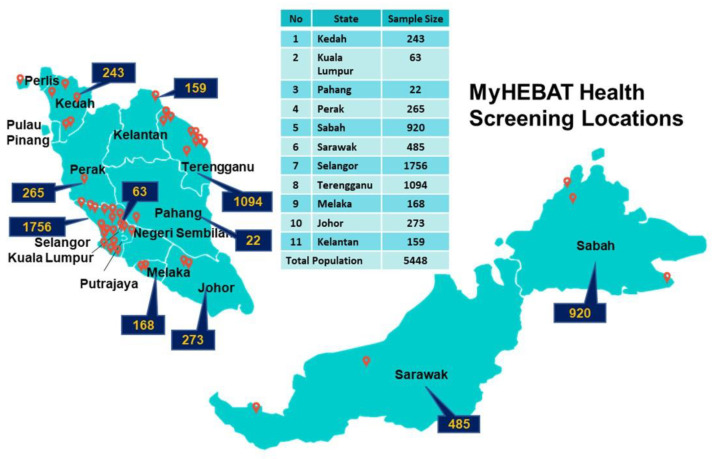
MyHEBAT health screening locations according to states. The figure indicates the locations and numbers of participants recruited throughout Malaysia.

The MyHEBAT study is an ongoing cross-sectional study which commenced in 2011 involving Malaysian communities from 11 states, representing all regions and federal territories, including rural and urban areas to ensure that the study covers a balanced sociodemographic Malaysian population. The states involved are Kedah, Kelantan, Kuala Lumpur, Pahang, Perak, Sabah, Sarawak, Selangor, Terengganu, Melaka and Johor. The total number of participants recruited from 2011 to 2018 for this study was 5448. Data collection was halted during the COVID-19 lockdowns which were imposed nationwide. As we moved into the endemic phase, data collection for MyHEBAT study is expected to resume until 2025. Figure 1 shows the number of participants which have been recruited from each state to date.

### 4.2. Sampling Methods: State and Site Selection

The MyHEBAT data collection included multiple races, tribes with different backgrounds; hence, the selected states represent the major ethnic groups in Malaysia. States in Peninsular Malaysia, namely, Kedah, Kelantan, Kuala Lumpur, Pahang, Perak, Selangor, Terengganu, Melaka and Johor, cover the main ethnic groups, which are the Malays, Chinese and Indians. The states in Eastern Malaysia, namely, Sabah and Sarawak, cover other ethnic minorities, which were classified as the indigenous group. In Sabah, the Bajau, Kadazan, Bugis, Suluk and Dusun are the main ethnic minorities, whereas in Sarawak, the main ethnic groups are Iban and Bidayuh.

### 4.3. Sampling Methods: Subject Recruitment

The subject recruitment followed a convenient sampling method through community health screening programmes. Announcements for the health screening were advertised by the local authority at least two weeks before the event day. At the screening desk, participants who agreed to participate were screened for eligibility based on the inclusion and exclusion criteria. The inclusion criteria were Malaysians aged between 15–75 years old; the exclusion criterion for the MyHEBAT-CRES study was pregnancy, and for the MyHEBAT-FH study, participants with secondary hypercholesterolaemia (hypothyroidism, chronic kidney disease, nephrotic syndrome and cholelithiasis) were excluded. This study was performed in concordance with the Declaration of Helsinki; ethical approval was obtained from the Institutional Research Ethics Committee (reference UiTM 600-IRMI [5/1/6]). Written informed consent was obtained from each participant before the enrolment into this study. For participants below the age of 18 years, consent was obtained from their legal guardians, and the questionnaire for the study was filled by their guardians. The study information sheet highlighted the rights of the participants to participate in the study voluntarily, and they could withdraw from the study at any time without penalty.

### 4.4. Definitions of Study Variables

Hypertension was defined as participants having systolic blood pressure (BP) ≥140 mmHg and/or diastolic BP of ≥90 mmHg or self-reported hypertension with/without taking anti-hypertensive medications [43]. Diabetes mellitus type 2 (DM) was defined as fasting plasma glucose (FPG) of ≥7.0 mmol/L and/or random plasma glucose (RPG) of ≥11.1 mmol/L, or self-reported DM with/without anti-diabetic medications [44].

Body mass index (BMI) was categorised following the Malaysia Clinical Practice Guidelines (CPG) on Management of Obesity, 2004 [45], where the BMI for underweight is <18.5 kg/m^2^, normal weight is 18.5–22.9 kg/m^2^, overweight is 23–27.4 kg/m^2^ and obesity is defined as having BMI of ≥27.5 kg/m^2^. Central obesity is defined as waist circumference (WC) measurement being ≥90 cm for males and ≥80 cm for females. Smoking was divided into three categories, namely, current smokers, non-smokers and ex-smokers [46]. Current smokers were individuals who were currently smoking at the time of recruitment or had smoked tobacco for the past month. Non-smokers were individuals who had never smoked, and ex-smokers were individuals who had quit smoking for more than five years.

The cut-off points for low HDL-C were <1.0 mmol/L for males and <1.2 mmol/L for females. The LDL-C cut-off points vary depending on the individuals’ CVD risk category group. Hypertriglyceridaemia (HTG) was defined as TG of >1.7 mmol/L. Hypercholesterolaemia (HC) was defined as individuals with TC of ≥5.2 mmol/L and/or taking lipid-lowering drug (LLD) and/or self-reported HC [46]. The summary of variable definitions is shown in Table 1.

### 4.5. Cardiovascular Disease Risk Stratification

Three cardiovascular risk tools were used to stratify the cardiovascular risk category for the participants. One of the risk tools used to stratify individuals for their 10-year cardiovascular risk assessment was the FRS-CVD, which is recommended by the Malaysian Clinical Practice Guidelines on the Primary and Secondary Prevention of Cardiovascular Disease, 2017 [36]. Individuals with FRS-CVD scores of <10% were categorised as low-risk, and individuals with FRS-CVD scores of 10–20% were categorised into moderate risk. Individuals with FRS-CVD scores of >20% were categorised as high risk [36,46]. Each individual was scored based on FRS, where CVD points were based on gender, age, HDL, TC, systolic blood pressure, smoking and diabetes status. Individuals with established CVD, CAD risk equivalent or severe hypercholesterolaemia were categorised as high-risk category regardless of their FRS score.

The ESC/EAS 2016 guideline is one of the established risk tools used in the European countries [39]. The participants of MyHEBAT study were classified using the ESC/EAS 2016 risk tool into low, moderate, high and very high-risk categories. All participants’ Systematic Coronary Risk Estimations (SCORE) of 10-year risk of fatal CVD were calculated based on gender, smoking, systolic blood pressure and cholesterol reading. Individuals with SCORE <1% were assigned to the low-risk group, whereas individuals with SCORE of ≥1% to <5% were classified into the moderate-risk group. Individuals in the high-risk category had SCORE of ≥5% to <10%, or total cholesterol > 8.0 mmol/L, or BP ≥ 180/110 mmHg, or had diabetes, or had moderate chronic kidney disease (glomerular filtration rate 30–59 mL/min/1.73 m^2^). Individuals in the very high-risk group had SCORE of ≥10%, or documented CVD, or DM (with target organ damage or with a major risk factor, such as of HPT, smoking, dyslipidaemia), or severe CKD (GFR < 30 mL/min/1.73 m^2^).

The ESC/EAS 2019 guideline is the latest established risk tool with similar SCORE requirements as the ESC/EAS 2016 guideline, but has several updated criteria [38]. The moderate-risk category has an additional optional criterion of diabetes < 10 years without other risk factors. The high-risk category has additional optional criteria of LDL-C > 4.9 mmol/L, or confirmed FH without other major risk factors, or diabetes (duration ≥ 10 years, or with an additional risk factor), or moderate chronic kidney disease (glomerular filtration rate < 30 mL/min/1.73 m^2^). The very high-risk category has an additional optional criterion of DM (with three major risk factors or type 1 DM > 20 years), or FH (with ASCVD or another major risk factor). The summary of CVD risk tools used in this study is shown in Table 2.

### 4.6. Familial Hypercholesterolaemia

For the MyHEBAT-FH Study, individuals were clinically diagnosed using four types of FH diagnostic criteria, namely, DLCN, SB, USMEDPAD and JFHMC. The summary of the diagnostic tools is shown in Table 3. The DLCN diagnostic categorised individuals into definite, probable, possible or unlikely FH [47]. In this study, the minimum baseline LDL-C levels for inclusion into this study for those with and without premature CAD were 4.0 and 4.9 mmol/L, respectively, as individuals with baseline LDL-C lower than these cut offs were categorised as unlikely FH [18]. Individuals who were taking statin but did not remember their baseline LDL-C had their untreated LDL-C estimated using LDL-C adjustment factors [48]. Individuals with DLCN scores of >8, ≥6–8 and ≥3–5 were defined as having definite, probable and possible FH, respectively. Both definite and probable FH were collectively called potential FH [49]. Individuals who did not meet the minimum score for possible FH were categorised as unlikely FH.

For the SB diagnostic criteria, participants were clinically diagnosed as definite, possible or unlikely FH [50,51]. Individuals with TC reading of >7.5 mmol/L as adults, or >6.7 mmol/L as children (age < 16 years), or LDL-C > 4.9 mmol/L as adults or LDL-C > 4.0 mmol/L as children, plus the presence of tendon xanthomas in a patient or relative (parents, siblings, children, grandparents, uncles or aunties), or confirmation of genetic mutation of LDL receptor, APOB or PCSK9, were categorised as definite FH. Individuals with severe hypercholesterolaemia (as above) in the presence of a family history of premature CAD (myocardial infarction at <60 years old among first degree relatives (parents, siblings or children), or <50 years old in second degree relatives (grandparents, aunts or uncles)) or family history of hypercholesterolaemia (as above) in first- or second-degree relatives, were categorised as possible FH. Individuals with none of the above combinations were categorised as unlikely FH.

For US MEDPED, the diagnostic criteria categorised participants into binomial outcome of FH or FH based on age and TC/LDL-C cut-off values of the participants. The TC cut-off values vary depending on the number of family members who were diagnosed with FH [52].

The JFHMC categorised individuals into having homozygous FH and heterozygous FH [53]. Homozygous FH was assigned to individuals having baseline TC ≥ 15.5 mmol/L, tendon xanthomata or nodular xanthoma; premature CAD during childhood; and parental family history of heterozygous FH. As for the heterozygous FH, individuals were given this diagnosis if they had ≥2 risk factors, namely, baseline LDL-C ≥ 4.7 mmol/L, or presence of tendon xanthomata or nodular xanthoma, or family history of FH or premature CAD (men < 55, women < 60 years old) within first- and second-degree relatives.

### 4.7. Study Procedures

Standardised and validated questionnaires for collecting participants’ general information, sociodemographic information, personal medical histories, family histories and lifestyles were distributed to the participants on site after written informed consent was obtained. The questionnaire recorded the sociodemographic data (age, gender, race, state, occupation background, household income, education background, marital status), menopause status, lifestyle (smoking, alcohol consumption), individual medical history [CAD, stroke, peripheral vascular disease (PVD), HC, current LLD therapy, hypertension and DM] and family medical history. Trained research assistants and health volunteers were on standby on-site to assist the participants in filling in the questionnaires. Anthropometric and biometric data were measured on-site following the method previously reported [54]. A pre-calibrated freestanding wall-mounted stadiometer (Seca 206, Seca GmbH & Co. KG.; Hamburg, Germany) was used to measure height and weight. The waist and hip circumference were measured using measuring tape. The BP of participants was measured using an automated BP monitor (Auto Blood Pressure Monitor HEM8712, Omron; Kyoto, Japan). Trained physicians were on site to perform physical examinations of FH clinical signs, such as the lipid stigmata, tendon xanthomata and corneal arcus, and gave on-site health consultation if necessary [18]. Secondary variables gained from the primary data, such as obesity category, BMI category, diagnosis of diabetes, hypertension, hyperlipidaemia, hypercholesterolaemia, dyslipidaemia, CVD risk and FH categories using various risk tools and clinical diagnostics, were included as well.

#### 4.7.1. Blood Collection and Laboratory Analysis

In total, 15 mL venous blood samples were collected in plain, fluoride oxalate and EDTA tubes from the health screening site. For blood samples in plain and fluoride oxalate tubes, the samples were processed on-site by centrifuging at 4000 rpm for 15 min to separate the serum or plasma for storage (−20 °C) until analysis. The serum lipid profile was analysed using an automatic analyser (COBAS Integra^®^ 400, Roche Holding AG; Basel, Switzerland) which includes TC, TG, HDL-C, lipoprotein (a) (Lp (a)) and glucose level analyses. The value of the LDL-C level was calculated using Friedewald equation, and participants of LLD had their baseline pre-treatment LDL-C level calculated from the conversion algorithm [18]. Upon completion of laboratory analysis for glucose level and fasting serum lipid profile, each participant’s CVD risk and FH categories were determined. For samples in EDTA tubes, the whole blood was kept at −20 °C. Only patients with positive clinical diagnosis of DLCN (definite, probable or possible FH) were selected for genetic testing. The whole blood samples from selected FH patients were subjected to a DNA extraction procedure and subsequently analysed for pathogenic FH gene mutations using a next-generation sequencing method.

#### 4.7.2. Statistical Analysis Plan

The MyHEBAT data collected from the year 2011 to date underwent a series of processes, including data acquisition, validation, harmonisation, transformation, analysis and reporting. All data were kept in electronic format, and the hardcopy questionnaires were kept in a secured archiving room for future reference. Data quality from the questionnaire and laboratory were evaluated for consistency and accuracy before being verified by at least two statisticians. Individuals with missing data will be excluded [55].

Descriptive statistics will be presented as frequencies and percentages for categorical variables. For numerical variables, mean ± standard deviation (SD) will be used for normally distributed data, and median with interquartile range (IQR) for non-normally distributed data. Chi-squared test or Fisher’s exact test will be used to analyse the associations between two groups. Simple logistic regression and multiple logistic regressions will be used to estimate the crude and adjusted odds ratio for the factors associated with the outcome variables of interest. A *p*-value of <0.05 will be considered statistically significant [56].

Analysis will be performed to compare FH detected using four clinical diagnostic criteria (DLCN, SB, USMEDPED and JFHMC). Sensitivity, specificity, positive, negative predictive and accuracy values will also be determined to compare the performances of the four clinical diagnostic criteria. Cohen’s kappa coefficient (κ) analysis will be used to measure inter-rater reliability and intra-rater reliability to evaluate the agreements among FH clinical diagnostic criteria. Similarly, the same test will be performed for the MyHEBAT-CRES study comparing the various CVD risk tools (FRS-CVD, ESC/EAS 2016 and 2019).

#### 4.7.3. Patient Management

**Figure 2:**
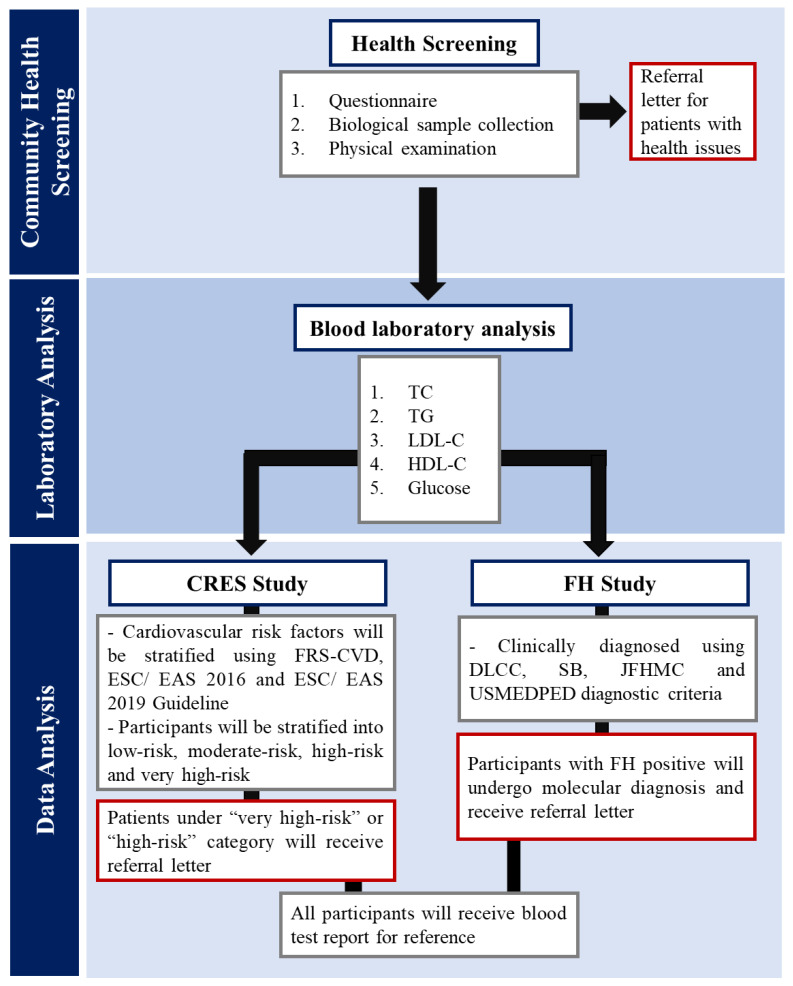
Overview of MyHEBAT study flow chart.

Participants were informed of their laboratory results for fasting serum lipid and glucose levels about one month after the date of enrolment by mail. Participants with imminent health issues, high-risk or very high-risk for CVD and positive for FH were identified and notified by means of referral letters advising them to seek further medical care at nearby healthcare centres. Figure 2 shows the flow chart for the MyHEBAT study.

## 5. Discussion

MyHEBAT study is a trailblazing national collaborative effort which gathers experts in specialised fields, including clinicians, specialists, epidemiologists and statisticians, to establish CVD risk factors and an FH database for the Malaysian population. The data were collected since 2011 through health screening across all states in Malaysia, and consist of detail information on sociodemographic factors (age, race, state, occupation background, education background, marital background), menopause status, lifestyle (smoking, alcohol consumption), individual medical history, family medical history and details of blood profiles (TC, TG, HDL-C, LDL-C, glucose). In addition, physical examinations such as corneal arcus, xanthelasma, weight, height, WC, HC, systolic and diastolic BP were recorded as well. Secondary variables gained from the primary data, such as obesity category, BMI category, diagnosis of diabetes, hypertension, hyperlipidaemia, hypercholesterolaemia, dyslipidaemia, CVD risk and FH categories using various risk tools and clinical diagnostics, will be available as well.

MyHEBAT database will provide understanding on the prevalence of CVD risk factors in Malaysia. Using the database, researchers will be able to study specific aspects of risk factors and their associations with specific sociodemographic variables (regions, ethnicities, socioeconomics classes, education), thereby understanding the pattern of the CVD risk factors among the Malaysian population. As mentioned earlier, participants’ CVD and FH risk categories were evaluated using multiple risk assessment and clinical diagnostic tools. The results from this study will provide a better understanding of the use of these tools in the Malaysian population. Targeted interventions can then be focused on those in the high-risk categories.

There are several potential impacts of the MyHEBAT study on the quadruple helix framework, namely, on academic research, government policy, industry and the society [57]. With regards to the impact on academia, MyHEBAT will provide extensive data on the prevalence and risk categorisation of CVD and FH to bridge the gaps in the literature. Output from this study will be published in scientific journals, books and conference presentations which will be beneficial to the government, industry and society.

MyHEBAT will also have impacts on policy makers and healthcare practitioners. Evidence from the MyHEBAT study will aid clinicians and specialists in identifying patients at high risk of CVD and offer preventive management. From the MyHEBAT database, patients with FH will be diagnosed early, and treatment with LLD and cascade screening of family members can be initiated earlier. Evidence from the MyHEBAT study could also be translated into clinical practice guidelines for healthcare providers and also guide policy makers to channel resources into those who are at high risk of CVD. Targeted management of those at high risk has been shown to be effective at reducing the healthcare burden and expenditure on healthcare [58]. Therefore, MyHEBAT offers a synergistic collaboration between researchers, healthcare providers and policy makers, translating evidence into practice and policies that will benefit the nation. The government has the constitutional authority to enact, implement and enforce policies to promote a healthy lifestyle in Malaysia through education, housing, transportation, the environment, zoning and taxes, safe streets, tobacco regulation and economic development [59]. Various studies have reported that Malaysians lacked knowledge on CVD risks or were unaware of the risks entirely [5,60,61,62,63]. This finding suggests the importance of multisectoral actions in educating Malaysian citizens at an early age about CVD risks and the importance of having a healthy lifestyle to prevent CVD through mass media, social media, schools and health facilities. The government could enforce smoking-free zones, increase taxes for alcohol and cigarette products, create safe lanes for cyclists and much more. In addition, the government could play a major role in providing more budgets for cardiovascular research, which will benefit the country and the nation [64].

The MyHEBAT study will also have an impact on the industry whereby the evidence could be used to support production of healthy functional food to prevent CVD [65,66]. Furthermore, Malaysians with diabetes, hypertension, dyslipidaemia and chronic kidney disease will need special diets in order to manage their CVD risk factors and to improve their overall vascular health [67,68]. Evidence from this study can also be used to promote physical activities. Various organisations should rally efforts to organise sporting events such as fun runs, cycling and swimming contests for the general public. Studies have reported that adopting a healthy lifestyle resulted in improved cardiovascular risk factors, reduced risk of CVD and reduced CVD-related mortality [69,70,71].

The pharmaceutical industry plays an essential role in producing medications or supplements to prevent, treat and manage diseases, including CVD. Over the years, the demand for dietary supplements has been increasing due to awareness of the importance of maintaining good health [72,73,74]. Therefore, producing safe and efficacious products to meet the Malaysian demands on specific health conditions is critical and beneficial for the pharmaceutical industry and its consumers [75,76]. Moreover, the medical device industry or diagnostic industry could utilise MyHEBAT data to develop specific diagnostic kits (e.g., genetic test kit), medical instruments (e.g., cholesterol-measuring device) or software applications (e.g., electronic CVD risk assessment tool) to ease the processes of diagnosis and treatment. Multiple studies have demonstrated that utilising software applications, artificial intelligence via deep or machine learning improved the diagnosis and treatment of CVD and its risk factors [77,78].

Last but not least, the immediate impact of MyHEBAT study for the society would be the participants receiving the free health check and consultation during and after the health screening programme, in which they received reports on their CVD risk and lipid profiles, and referral letters for participants who need additional follow-up treatment. Furthermore, through MyHEBAT, the participants were informed of their health status which made them aware of whether they have CVD risk or were at the borderline of having the risk. This is essential, as several studies have reported that Malaysians have poor awareness about their risks, diseases, treatment and medication, which could lead to poor control of their CVD risk factors [5,60,61,79,80]. Overall, evidence from the MyHEBAT study will bridge the gaps in the literature and guide academics, policy makers and the industry in their collaborative efforts to battle CVD risk, morbidity and mortality.

### Strengths and Limitations

The primary strength of the MyHEBAT study is that this study recruited a relatively large number of participants from various regions, covering almost all states in Malaysia. Therefore, it gives a good representation of the Malaysian population. Secondly, as compared to the NHMS, which only reported limited types of lipid biomarkers, the MyHEBAT study analysed complete blood profiles, which included TC, TG, HDL-C, Lp(a) and glucose. Despite the LDL-C being obtained through the Friedewald equation, the complete blood profiles provided more comprehensive data analysis and data interpretation. In addition, the MyHEBAT-FH study has also recruited the largest number of FH patients in Malaysia thus far. This study will report FH prevalence from multiple states in Malaysia, which will be representative of the Malaysian population. While some countries did not include relevant clinical history and physical examination for detection of FH clinical features as part of their FH screening protocols [81,82], the MyHEBAT-FH study have included them for better accuracy of FH diagnosis.

This study has several limitations, which include potential selection bias during patient recruitment due to the convenient sampling method. However, a large sample size involving multiple sites, covering almost all states in Malaysia, may have remedied this potential selection bias. The second limitation for this study is recall bias, as data were collected using interviews. Participants may not give accurate information regarding their family histories, particularly of premature CAD and hypercholesterolaemia among family members.

## 6. Conclusions

MyHEBAT study is a national-level epidemiological programme, combining joint efforts from multi-disciplinary medical specialties, which have been assembled to tackle Malaysian health and well-being issues closely related to CVD risks and FH. This paper described the details of the MyHEBAT study protocol. The MyHEBAT study has foreseeable positive impacts for academia, government, industry and society in the long run. This study has prompted academics and researchers to utilise their expertise in developing the database. Findings from this study will be translated into new and meaningful knowledge which will bridge the knowledge gap in the literature. Furthermore, the data and knowledge from this study could guide policy change and resource allocation by the government. This finding could be used to initiate the government to enact or implement laws to encourage healthy lifestyles. Additionally, the industry could use the study findings to support them to manufacture safe and efficacious health products to meet the needs of Malaysian society. Ultimately, the MyHEBAT study will promote collaborative efforts between the academia, healthcare providers, policy makers, society and industries in battling the CVD epidemic in order to reduce complications, reduce hospitalisations, reduce healthcare costs, prolong life and increase quality of life towards becoming a healthier nation.

## Figures and Tables

**Table 1:** Summary of variables definition.

No.	Abbreviation	Variables	Description	Type of Data	Reference
1	HPT	Hypertension	SBP ≥ 140 mmHg and/or DBP ≥ 90 mmHg or self-reported of having HPT with/without taking anti HPT medication	Nominal	[43]
2	DM	Diabetes mellitus type 2	FPG ≥ 7.0 mmol/L or RPG ≥ 11.1 mmol/L or self-reported of having DM with/without anti DM medication.	Nominal	[44]
3	BMI	Body Mass Index	1. Underweight (BMI < 18.5 kg/m^2^) 2. Normal (18.5 ≤ BMI ≥ 22.9) 3. Overweight (23 ≤ BMI ≥ 27.4 kg/m^2^) 4. Obese (BMI of ≥27.5)	Ordinal	[45]
4	CO	Central Obesity	Waist circumference ≥ 90 cm for males and ≥80 cm for females.	Nominal	[45]
5	Smoking	Smoking	1. Smoker 2. Non-smoker 3. Ex-smoker	Nominal	[46]
6	Low HDL-C	Low high-density lipoprotein cholesterol	Male < 1.0 mmol/L, female < 1.2 mmol/L	Nominal	[46]
7	High LDL-C	High low-density lipoprotein cholesterol	Low-risk group > 3.4 mmol/L Moderate-risk group > 2.6 mmol/L High-risk group > 2.6 mmol/L Very high-risk group > 1.8 mmol/L	Nominal	[46]
8	HTG	Hypertriglyceridaemia	TG > 1.7 mmol/L	Nominal	[46]
	HC	Hypercholesterolaemia	TC ≥ 5.2 mmol/L and/or taking lipid-lowering drug and/or self-reported of having HC	Nominal	[46]

Systolic blood pressure = SBP; diastolic blood pressure = DBP; fasting plasma glucose = FPG; random plasma glucose = RPG; TG = triglyceride; TC = total cholesterol.

**Table 2:** Summary of three cardiovascular disease risk scores, Framingham Risk Score—Cardiovascular Disease (FRS-CVD), European Society of Cardiology/European Atherosclerosis Society (ESC/EAS) 2016 and ESC/EAS 2019 Guidelines that were used to stratify the participants into low, moderate, high and very-high risk.

	FRS-CVD 2008	ESC/EAS 2016	ESC/EAS 2019
**Low**	<6% of 10 year-risk CVD prediction.	SCORE < 1% for 10-year risk of fatal CVD	SCORE < 1% for 10-year risk of fatal CVD.
**Moderate**	6–20% of 10 year-risk CVD prediction.	SCORE is ≥1% and <5% for 10-year risk of fatal CVD	Type 1 DM < 35 years; type 2 DM < 50 years) with DM duration < 10 years, without other risk factors. SCORE ≥ 1 % and <5% for 10-year risk of fatal CVD.
**High**	20% of 10 year-risk CVD prediction. CVD equivalent (CHD, cerebrovascular events, peripheral artery disease and heart failure).	Elevated single risk factors, in particular cholesterol > 8 mmol/L or BP ≥ 180/110 mmHg. DM. Moderate CKD (GFR 30–59 mL/min/1.73 m^2^). SCORE ≥ 5% and <10% for 10-year risk of fatal CVD.	Elevated single risk factors, TC > 8 mmol/L, LDL-C > 4.9 mmol/L, or BP ≥ 180/110 mmHg. Have FH without other major risk factors. DM duration ≥ 10 years or another additional risk factor. Moderate CKD (eGFR 30–59 mL/min/1.73 m^2^). SCORE ≥ 5% and <10% for 10-year risk of fatal CVD.
**Very High**	N/A	Documented CVD DM with target organ damage or with a major risk factor either smoking, hypertension or dyslipidaemia. Severe CKD (GFR < 30 mL/min/1.73 m^2^). SCORE ≥ 10% for 10-year risk of fatal CVD.	Documented CVD DM with target organ damage or three major risk factors or Type 1 DM > 20 years. Severe CKD (eGFR < 30 mL/min/1.73 m^2^). SCORE ≥ 10% for 10-year risk of fatal CVD. FH with ASCVD or with another major risk factor.

CVD = cardiovascular disease; CHD = coronary heart disease; SCORE = System for Cardiac Operative Risk Evaluation; BP = blood pressure; DM = diabetes mellitus; CKD = chronic kidney disease; GFR = glomerular filtration rate; TC = total cholesterol; LDL-C = low-density lipoprotein cholesterol; ASCVD = atherosclerotic cardiovascular disease.

**Table 3:** Summary of all FH diagnostic criteria tools and their related variables used in MyHEBAT-FH study.

	DLCN	SB	JFHMC	USMEDPED
Diagnostic outcome categories	Definite Probable Possible Unlikely	Definite Possible Unlikely	Yes No	Yes No
Lipid data used	LDL-C	TC or LDL-C	TC or LDL-C	TC or LDL-C
Genetics evaluation	Yes	Yes	No	No
Personal history of PCAD	Yes	No	No	No
Family history PCAD	Yes	Yes	Yes	No
Family history of hypercholesterolaemia	Yes	Yes	No	No
Family History of FH	No	No	Yes	Yes
Physical examination of corneal arcus	Yes	No	No	No
Physical examination of tendon xanthomata	Yes	Yes	Yes	No

FH = familial hypercholesterolaemia; PCAD = premature coronary artery disease; DLCN = Dutch Lipid Clinic Network Criteria; SB = Simon Broome Registry Diagnostic Criteria; JFHMC = Japanese FH Management Criteria; USMEDPED = US Make Early Diagnosis to Prevent Early Deaths; TC = total cholesterol; LDL-C = low-density lipoprotein cholesterol.

## Data Availability

Not applicable.

## Abbreviations

APOB	Apolipoprotein B
ASCVD	Atherosclerotic cardiovascular disease
BP	Blood pressure
BMI	Body mass index
CVD	Cardiovascular disease
CO	Central obesity
CPG	Clinical Practice Guidelines
CAD	Coronary artery disease
CRES	Coronary Risk Epidemiological Study
DM	Diabetes mellitus
DNA	Deoxyribonucleic acid
DLCN	Dutch Lipid Clinic Network Criteria
EDTA	Ethylenediaminetetraacetic acid
ESC/EAS	European Society of Cardiology/European Atherosclerosis Society
FH	Familial hypercholesterolaemia
FPG	Fasting plasma glucose
FRS-CVD	Framingham Risk Score Cardiovascular Disease
HDL-C	High-density lipoprotein cholesterol
HTG	Hypertriglyceridaemia
HC	Hypercholesterolaemia
HPT	Hypertension
IQR	Interquartile range
JFHMC	Japanese Familial Hypercholesterolaemia Management Criteria
Lp (a)	Lipoprotein (a)
LLD	Lipid lowering drug
LDL-C	Low-density lipoprotein cholesterol
MyHEBAT	Malaysian Health and Wellbeing Assessment
NHMS	National Health and Morbidity Survey
NSP-NCD	National Strategic Plan for Non-Communicable Diseases
PVD	Peripheral vascular disease
PCSK9	Proprotein convertase subtilisin/kexin type 9
RPG	Random plasma glucose
SB	Simon Broome’s Register Criteria
SD	Standard deviation
SCORE	Systematic Coronary Risk Estimation
TC	Total cholesterol
TG	Triglyceride
USMEDPED	United States Make Early Diagnosis to Prevent Early Deaths Criteria
WC	Waist circumference

## Appendix A. Malaysian HEalth and Well-Being AssessmenT (MyHEBAT) Research Investigators

**UNIVERSITI TEKNOLOGI MARA (UiTM):** Hapizah Nawawi (I-PPerForM, Faculty of Medicine and Al-Sultan Abdullah Hospital), Anis Safura Ramli (I-PPerForM and Faculty of Medicine), Suraya Abdul Razak (I-PPerForM and Faculty of Medicine), Noor Alicezah Mohd Kasim (I-PPerForM, Faculty of Medicine and Al-Sultan Abdullah Hospital), Thuhairah Hasrah Abdul Rahman (I-PPerForM, Faculty of Medicine and Al-Sultan Abdullah Hospital), Effat Omar (I-PPerForM, Faculty of Medicine and Al-Sultan Abdullah Hospital), Noor Shafina Mohd Nor (I-PPerForM, Faculty of Medicine and Al-Sultan Abdullah Hospital), Alyaa Al-Khateeb (I-PPerForM and Faculty of Medicine), Suhaila Abd Muid (I-PPerForM and Faculty of Medicine), Mansharan Kaur Chainchel Singh (I-PPerForM, Faculty of Medicine and Al-Sultan Abdullah Hospital), Yung-An Chua (I-PPerForM), Nurul’Ain Abu Bakar (I-PPerForM), Aimi Zafira Razman (I-PPerForM), Sukma Azureen Nazli (I-PPerForM), Amirah Mohd Ariff (I-PPerForM), Fadlullah Jili Fursani Kemrry (I-PPerForM), Mohd Yusmiaidil Putera Mohd Yusof (I-PPerForM and Faculty of Dentistry), Zaliha Ismail (Faculty of Medicine), Norashikin Mohd Ranai (Faculty of Medicine and Al-Sultan Abdullah Hospital), Ahmad Taufik Jamil (Faculty of Medicine and Al-Sultan Abdullah Hospital), Ahmad Bakhtiar Md Radzi (Faculty of Medicine and Al-Sultan Abdullah Hospital), Khairul Shafiq Ibrahim (Faculty of Medicine and Al-Sultan Abdullah Hospital), Salmi Razali (I-PPerForM, Faculty of Medicine and Al-Sultan Abdullah Hospital), Muthukkaruppan Annamalai (Faculty of Computer Science and Mathematics), Marshima Mohd Rosli (I-PPerForM and Faculty of Computer Science and Mathematics), Ahmad Zia Ul-Saufie (Faculty of Computer Science and Mathematics), Siti Hamimah Sheikh Abdul Kadir (I-PPerForM and Faculty of Medicine), Sazzli Shahlan Kasim (I-PPerForM, Al-Sultan Abdullah Hospital, CaVaLRI and Faculty of Medicine), Hasidah Abdul Hamid (Faculty of Medicine), Siti Munira Yasin (Faculty of Medicine), Teh Lay Kek (iPROMISE and Faculty of Pharmacy); **UNIVERSITY OF MALAYA (UM):** Mohd Amin Jalaludin (Faculty of Medicine and University Malaya Medical Centre); **UNIVERSITI SULTAN ZAINAL ABIDIN (UniSZA):** Ahmad Zubaidi Abdul Latif (Faculty of Medicine and UnisZA Teaching Hospital); **NATIONAL HEART INSTITUTE (IJN):** Azhari Rosman, Abdul Rais Sanusi; **NATIONAL DEFENCE UNIVERSITY OF MALAYSIA (UPNM):** Muhamed T Osman (Faculty of Medicine and Defence Health); **UNIVERSITI KUALA LUMPUR (UniKL):** Osman Ali (Royal College of Medicine Perak).
